# Supplementary material for: Integrative Landscape of Dry AMD Pathogenesis, Models, and Emerging Therapeutic Strategies
Source: Int J Mol Sci. 2025 Dec 24;27(1):202. doi: 10.3390/ijms27010202 (PMC12786248; doi:10.3390/ijms27010202)
Supplement: Supplementary file 1 [file ijms-27-00202-s001.zip › ijms-4029035-supplementary.pdf]

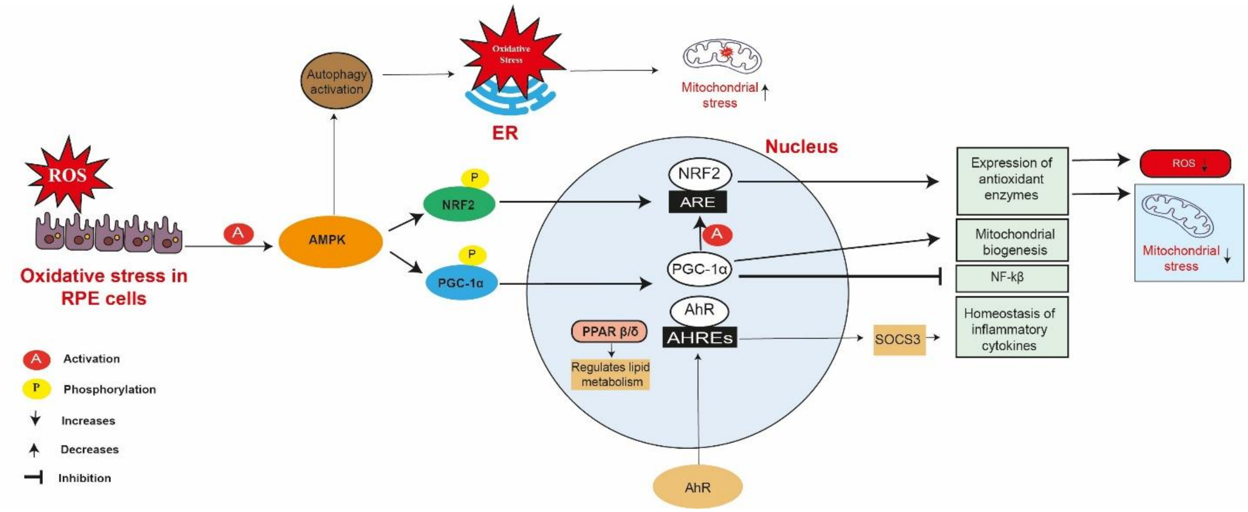

Supplementary Figure S1. Schematic of oxidative stress-mediated signaling pathways in RPE cells. Oxidative stress in RPE cells activates the AMPK signaling pathway through AMPK phosphorylation. This activation promotes autophagy, exacerbates oxidative stress in the endoplasmic reticulum (ER), and increases mitochondrial oxidative stress. AMPK also phosphorylates NRF2 and PGC-1α, promoting their nuclear translocation and the subsequent activation of genes involved in mitochondrial biogenesis, antioxidant enzymes expression, and inhibition of NF-kβ. This results in reduced transcription of proinflammatory cytokines and chemokines. PPARβ/δ modulates lipid metabolism and enhances cellular defenses by reducing oxidative stress. In parallel, the aryl hydrocarbon receptor (AhR) translocates to the nucleus, where it forms a complex with aryl hydrocarbon response elements (AHREs) to upregulate SOCS3 expression, maintaining cytokine homeostasis and regulating inflammatory signaling. ROS, reactive oxygen species; AMPK, AMP-activated protein kinase; PGC-1α, peroxisome proliferator-activated receptor gamma coactivator 1-alpha; NF-kβ, nuclear factor kappa-light-chain-enhancer of activated βcells; NRF2, nuclear factor erythroid 2-related factor 2; ARE, antioxidant response elements; ER, endoplasmic reticulum; PPARβ/δ, peroxisome proliferator-activated receptors; AhR, aryl hydrocarbon receptor; AHREs, aryl hydrocarbon response elements; SOCS3, suppressor of cytokine signaling 3.
